# Differential diagnosis of Hashimoto’s thyroiditis: The importance of pathological anatomy

**DOI:** 10.14440/jbm.2024.0139

**Published:** 2025-08-07

**Authors:** Hernando Vargas-Uricoechea, Ivonne Meza-Cabrera, Alejandro Castellanos-Pinedo, Karen Urrego-Noguera, María V. Pinzón-Fernández

**Affiliations:** 1 Department of Internal Medicine, Faculty of Health Sciences, University of Cauca, Popayán, 190001 Cauca, Colombia; 2 Department of Pathology, Faculty of Health Sciences, University of Cauca, Popayán, 190001 Cauca, Colombia; 3 Department of Internal Medicine, Faculty of Medicine, University of Sinú, Hospital San Jerónimo, Montería, 230001 Córdoba, Colombia; 4 Department of Internal Medicine, University of Cauca, Popayán, 190001 Cauca, Colombia

**Keywords:** Thyroiditis, Hashimoto, Autoimmunity, Histopathology

## Abstract

**Background:**

Hashimoto’s thyroiditis (HT) represents a complex, multifactorial autoimmune disease characterized by a broad spectrum of clinical and imaging presentations. These manifestations tend to be clinically perplexing, necessitating additional tests. In cases where a malignancy is suspected or obstructive symptoms are present, surgical management may be required. Histopathologically, HT is hallmarked by significant lymphocytic infiltration of the thyroid gland, typically with prominent germinal centers, small lymphocytes, centrocytes, centroblasts, plasma cells, and occasionally immunoblasts. The degree of fibrosis varies, giving the gland a lobulated appearance and, in some cases, resulting in a fibrous variant, which, biochemically, is usually accompanied by hypothyroidism.

**Objective:**

This review provided a diagnostic and differential approach to HT, highlighting the utility of histopathological examination.

**Conclusion:**

Histopathological analysis helps broaden the diagnostic and therapeutic horizon in individuals with HT.

## 1. Introduction

Hashimoto’s thyroiditis (HT) is an autoimmune disease characterized by conspicuous destruction of thyroid cells. This process results from various cell-mediated immune responses and an increased synthesis and secretion of thyroid autoantibodies (Abs).^1^,^2^ HT is the main cause of hypothyroidism in iodine-sufficient regions across the globe. Its global prevalence varies, depending on multiple factors (*e*.*g*., socioeconomic status, environmental exposures, and genetics) and is estimated to stand at 4.8–25.8% in men and 0.9–7.9% in women.^1^-^6^

Diagnosis of HT is often challenging and may not be established until the disease has reached an advanced stage. The most common laboratory findings include elevated levels of thyrotropin, with either low or normal levels of free thyroxine – indicating overt or subclinical hypothyroidism, respectively – along with the presence of Abs directed against thyroid peroxidase (TPOAb) or thyroglobulin (TgAb).^1^,^3^-^6^

Patients with HT and goiter (diffuse or nodular), especially when there is an obstructive component involving the esophagus or trachea, may present with clinical manifestations, such as dysphagia, sialorrhea, respiratory difficulty, stridor, and dysphonia. These patients are often evaluated with fine needle aspiration cytology when thyroid nodules are suspicious for malignancy on ultrasound (US), and may eventually undergo thyroidectomy.^7^,^8^

Histopathological findings in HT include diffuse and dense infiltration of small lymphocytes, although plasma cells, histiocytes, and giant cells may also be present. However, the hallmark feature of HT is the presence of lymphoid follicles of different sizes, associated with follicular atrophy and destruction of thyroid follicles.^8^ Conventional treatment for hypothyroid patients typically involves levothyroxine (LT4), which generally provides symptomatic relief.^9^

Although multiple classification systems and scoring methods exist (particularly from cytological and histopathological perspectives) for classifying thyroid lesions as benign or malignant, there is no universal consensus for diagnosing benign thyroid conditions, such as HT. While several accepted histopathological and/or cytological criteria are available, their interpretation can vary.^10^ Moreover, despite descriptions based on surgical specimens, gaps remain in clinicians’ understanding of histopathological findings. Misinterpretation of such reports may cause confusion and potentially delay the diagnosis and management of affected patients.

This review summarizes and discusses the most relevant clinical and histopathological aspects of HT, along with its differential diagnoses.

## 2. Methodology

**Figure 1:**
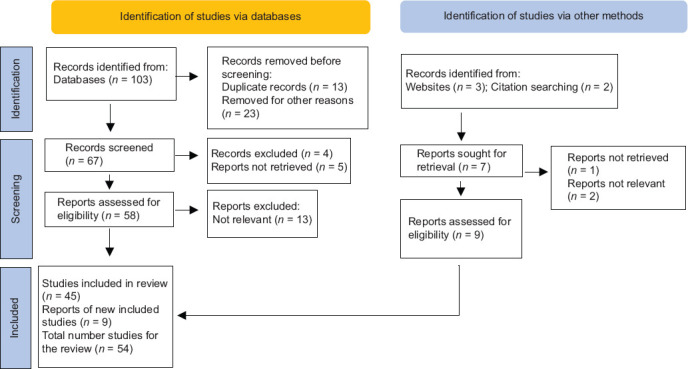
Preferred Reporting Items for Systematic Reviews and Meta-Analyses flow diagram: Method for article selection.

A literature search was conducted in the following databases: PubMed/MEDLINE, ProQuest, Scopus, BIOSIS, and Web of Science. The search covered articles published from January 1984 to December 2024. The following keywords were used: “Hashimoto’s thyroiditis,” “Hashimoto’s disease,” “chronic lymphocytic thyroiditis,” and “autoimmune thyroiditis.” Clinical trials, review articles, systematic reviews, meta-analyses, and studies describing the histopathological characteristics of this condition were included, while duplicate studies were excluded. The identification and selection of studies were carried out independently by two authors (HV-U and IM-C). Data were extracted using a standardized template within a predefined Excel form. In cases where discrepancies arose, the authors collaboratively conducted a second round of data extraction to validate the accuracy of the information. Only articles written in English were considered (Figure 1).

## 3. Historical aspects of HT

Hakaru Hashimoto (1881–1934) was born in a town in Mie prefecture, located southeast of Kyoto and east of Nara and Osaka, Japan. In 1912, Hakaru published an article entitled “Notes on the Knowledge of Lymphomatous Changes in the Thyroid Gland (Struma Lymphomatous).” The article described the histopathological changes in thyroid tissue from four adult women who had undergone partial thyroidectomy, initially differentiating it with Graves–Basedow disease (GBD), which was ruled out based on the clinical features observed at the time.^11^-^14^

In addition, he analyzed what were then recognized as diseases characterized by an “excess of lymphatic tissue,” which were classified as “inflammatory” (although they were not associated with the classic signs of acute inflammation). In fact, he suggested that these entities were more accurately described as chronic inflammatory disorders, further ruling out chronic infections, such as tuberculosis or syphilis.^14^,^15^

**Figure 2:**
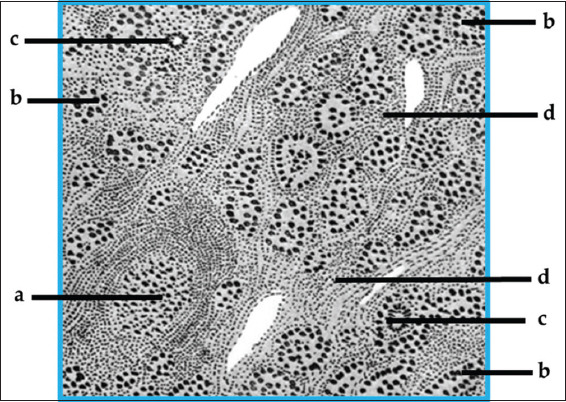
Histopathological characteristics of “struma lymphomatous” as described by Hakaru Hashimoto in 1912 Notes: a: Lymphoid follicle; b: Degenerated thyroid follicle; c: Giant cell; d: Hyperplastic interstitium with round cell infiltration. Source: Adapted from Caturegli *et al*.^12^

He then compared the findings of his patients with those of Riedel’s thyroiditis (RT) and found some histological similarities, although he noted that patients with RT exhibited more extensive and aggressive fibrosis than his own cases. Based on these observations, Hashimoto concluded that the two diseases were distinct entities and that what he was describing was, in fact, a new disease. The features he identified in the thyroid tissue of his four patients included: infiltration of lymphoid and plasma cells, formation of lymphoid follicles with germinal centers, fibrosis, degeneration of thyroid epithelial cells, and luminal leukocytes (Figure 2).^12^,^14^

Initially, the findings were not convincing enough to be considered a new disease, and some researchers and scholars regarded them as merely a type of chronic thyroiditis. In the subsequent years, the disease still was not recognized as a distinct entity. For example, British pathologist George Scott Williamson described similar results, which he termed “lymphadenoid goiter,” without referencing or acknowledging Hakaru’s earlier work.^12^-^14^

Later, in the 1930s, Allen Graham clearly and justifiably recognized that the findings described by Hashimoto represented a new disease entity, distinct from RT. Shortly thereafter, Hashimoto’s work was cited in the second edition of Cecil Joll’s textbook on thyroid diseases, in a chapter titled *Pathology, Diagnosis, and Treatment of Hashimoto’s Disease (Struma Lymphomata)*. The term became widely accepted following the Third International Conference on Goiter, held in Washington, D.C. (United States of America), in 1938.^13^-^16^

In 1956, Noel Rose and Erns Witebsky^17^ demonstrated that immunizing rabbits with thyroid extracts induced histological changes in the gland similar to those observed in HT. In addition, they detected the presence of TgAb in the sera of these rabbits. In the same year, Deborah Doniach and her team^18^ successfully purified a TgAb from the serum of patients with HT, concluding that these individuals had an immune response directed against thyroglobulin, and proposed that HT was an organ-specific autoimmune disease.

Subsequently, in a “Letter to the Editors” published in *The Lancet* in the early 1960s, Doniach^17^ wrote: “Our interest in autoimmune thyroiditis led us to commemorate the 50^th^ anniversary of Hashimoto’s description of struma lymphomata. Perhaps your readers would like to know more about this man.”

This letter is considered a turning point in the global recognition of Hakaru Hashimoto’s findings, marking the beginning of widespread use of terms, such as HT, Hashimoto’s disease, chronic lymphocytic thyroiditis, and autoimmune thyroiditis, in the medical literature.

## 4. Histopathological features of the normal thyroid

### 4.1. Macroscopic findings

**Figure 3:**
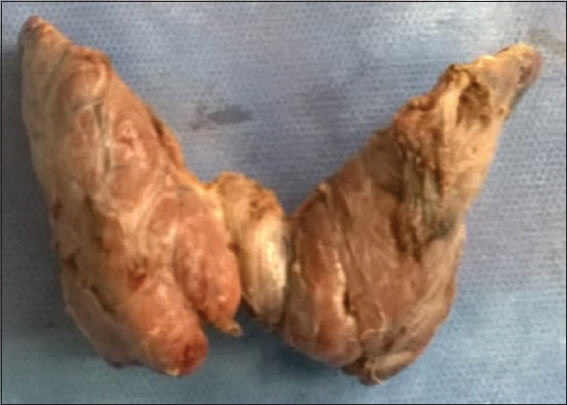
Normal thyroid; macroscopic image Note: Its characteristic butterfly shape, with the isthmus centrally located, joining the right and left lobes. The surface is slightly nodular and brownish-pink. Source: Authors’ data.

Macroscopically, the thyroid has a brownish-pink, slightly nodular, and vascularized surface. It is elastic in consistency when sectioned and is surrounded by a thin layer of connective tissue known as the perithyroid fascia, which thickens posteriorly to attach to the cricoid cartilage^19^ (Figure 3).

### 4.2. Microscopic findings

In a normal thyroid gland, a thin capsule of connective tissue penetrates the lobes, subdividing the gland into irregular lobular units. Each lobe contains groups of follicles, which are the structural and functional units of the gland. Each follicle is surrounded by a thin stroma of connective tissue rich in fenestrated capillaries, sympathetic nerve fibers, and lymphatic vessels.^19^,^20^

**Figure 4:**
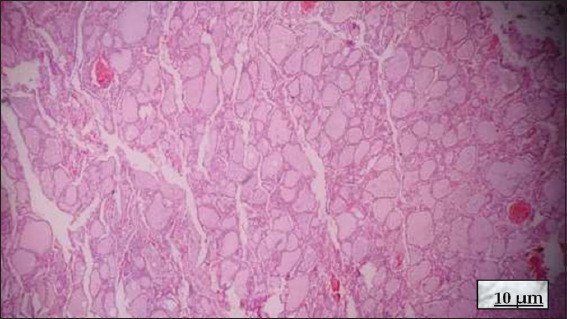
Normal thyroid; hematoxylin and eosin staining; 10× magnification, scale bar = 10 μm. Thyroid follicles of regular size are observed, with some smaller ones. Homogeneous eosinophilic colloid is present inside the follicles (toward 4, 5, and 10, clockwise), and three small vessels with erythrocytes are observed. Source: Authors’ data.

The follicular epithelium is a simple epithelium composed of low columnar, cuboidal, or flat cells, depending on the level of activity of the follicle. The colloid stains homogeneously pink with hematoxylin and eosin (HE), while the follicular cells appear bluish due to basophilic nuclei^19^,^20^ (Figure 4).

## 5. Histopathological features in HT

### 5.1. Macroscopic findings

**Figure 5:**
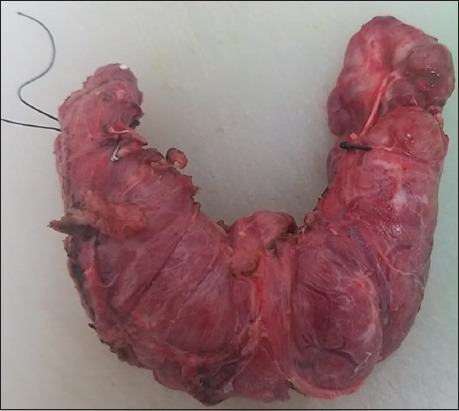
Hashimoto’s thyroiditis and diffuse goiter. Macroscopic image showing an enlarged gland with a light brown, nodular, and congested surface. Source: Authors’ data.

**Figure 6:**
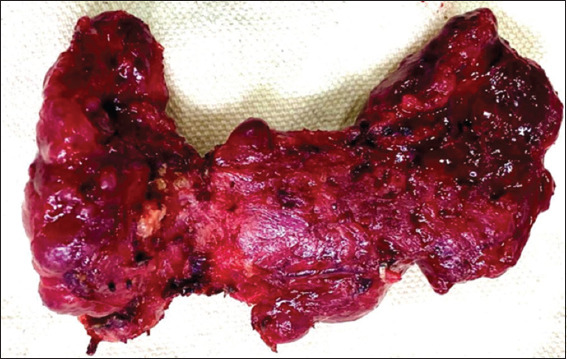
Hashimoto’s thyroiditis and multinodular goiter. Macroscopic image showing an enlarged gland with a reddish surface, areas of multifocal hemorrhage, and nodular areas characteristic of this pathology. Source: Authors’ data.

HT is characterized by a nodular or diffuse enlargement of the gland (two to three times its normal size), often involving the pyramidal lobe, and can weigh more than 200 g. Upon sectioning, lobules with varying degrees of fibrosis are observed, displaying a yellowish or light-brown coloration. Nodules, cysts, and bleeding areas may also be present. In long-standing HT, bands of fibrous tissue may form; this fibrous variant is firm, with a cut surface resembling multinodular liver cirrhosis^21^ (Figures 5 and 6).

### 5.2. Microscopic findings

**Figure 7:**
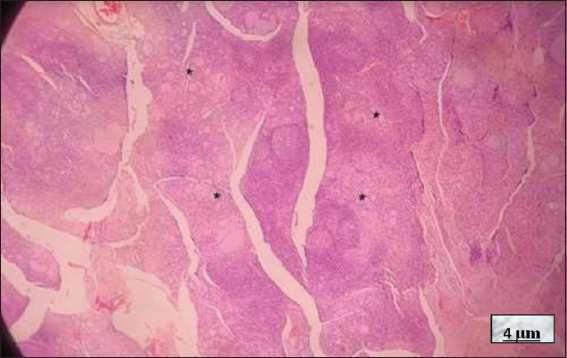
Hashimoto’s thyroiditis. Hematoxylin and eosin staining; 4× magnification, scale bar = 4 μm. Note: The predominantly small thyroid follicles (black asterisks), containing little colloid, surrounded by an abundant lymphocytic infiltrate. The infiltrate forms lymphoid follicles with prominent germinal centers. The white spaces are cutting artifacts, referred to as the “crackling effect,” caused by the dense lymphocytic infiltrate. Source: Authors’ data.

**Figure 8:**
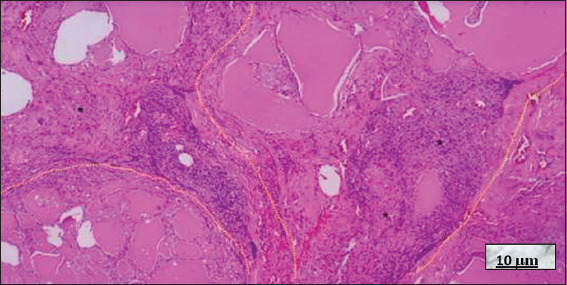
Fibrotic variant of Hashimoto’s thyroiditis. Hematoxylin and eosin staining; 10× magnification, scale bar = 10 μm. Note the marked fibrosis dividing the gland into lobes, with a diffuse lymphocytic infiltrate located between the fibrotic bands (yellow dotted line). A nodule of lymphoid follicles is visible in the lower left area, and destroyed thyroid follicles (black asterisks) are present in the lower right and middle left areas, within a fibrotic band. Source: Authors’ data.

Microscopically, HT shows significant lymphocytic infiltration of the thyroid gland, usually with prominent germinal centers, including small lymphocytes, centrocytes, centroblasts, plasma cells, and occasionally immunoblasts. Thyroid follicles are often trapped within the lymphocytic infiltrate (most of them atrophic) and may even be destroyed, leaving scattered thyrocytes that mimic multinucleated giant cells. Metaplastic changes, such as squamous or oncocytic metaplasia, may also be observed. Fibrosis is variable, giving the gland a lobulated appearance, and in some cases, may be severe, resulting in the fibrous variant, which is characterized by a lower lymphocyte population and more extensive squamous metaplasia^21^-^24^ (Figures 7 and 8).

**Figure 9:**
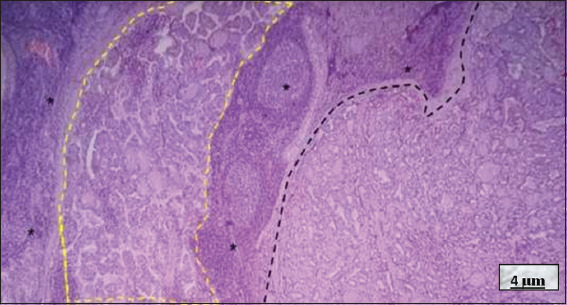
Hashimoto’s thyroiditis (HT) coexisting with papillary thyroid carcinoma (PTC). Hematoxylin and eosin staining; 4× magnification, scale bar = 4 μm. In the center and to the left of the image, note the dense lymphocytic infiltrate forming lymphoid follicles with germinal centers (black asterisks), typical of HT. On the left side, small papillary projections are visible, characteristic of PTC (yellow dotted line). On the right side, medium-sized and small thyroid follicles without cellular atypia are observed (black dotted line). Source: Authors’ data.

Occasionally, thyrocytes may show reactive atypia (consisting of irregular nuclear membranes, intranuclear rods, and even nuclear clearing); these findings are very similar to those observed in papillary thyroid carcinoma (PTC) (Figure 9). In fact, the association between HT and PTC is frequently described.^21^-^24^

## 6. Differential diagnosis of HT

### 6.1. Diagnosis based on clinical, laboratory, and imaging findings

HT may exhibit features that overlap with other diseases, rendering differential diagnosis particularly challenging. The main conditions to consider include, among others: De Quervain’s (or subacute) thyroiditis (DQT), GBD, primary thyroid lymphoma (PTL), oncocytic adenoma (OA) – previously known as Hürthle cell adenoma – RT, and PTC.^22^,^25^

Clinically, the differential diagnosis of HT is made in the vast majority of cases by means of a thorough clinical history, supported by laboratory findings, US, and radionuclide imaging (RI) of the thyroid using either Tc-99 or 123/131-I. Fine needle aspiration cytology and histopathological studies are rarely required, except in patients with suspected thyroid cancer, hyperfunctioning nodules (single or multiple), or when there is a significant compressive effect on adjacent structures (*e*.*g*., in cases of dysphagia or dysphonia due to compressive goiters), or when a rapid euthyroid state must be achieved in patients with GBD.^2^,^25^

In this context, DQT is a form of thyroiditis usually preceded by viral involvement (*e*.*g*., respiratory, gastrointestinal). It is not considered a type of autoimmune thyroid disease, although a significant proportion of patients may present with TPOAb and TgAb.^26^

It is often difficult to distinguish clinically between these two types of thyroiditis, especially during the hyperthyroidism phase (*e*.*g*., when HT initially presents with hyperthyroidism – Hashitoxicosis). However, in many cases, the patient’s history and clinical presentation provide clear clues. HT often manifests with a protean and more insidious clinical course, while DQT is typically more specific, with a rapid onset of spontaneous pain or pain on palpation of the thyroid, accompanied by fever, dysphagia, and general malaise in more than 90% of cases. This is usually associated with elevated levels of inflammatory markers, such as erythrocyte sedimentation rate and C-reactive protein.^26^,^27^

Thyroglobulin and TPOAb are virtually always present in HT, whereas in DQT, their prevalence is variable. In DQT, thyroid US typically shows a predominantly hypovascular pattern, and RI of the thyroid reveals very low uptake of the radiotracers used.^25^-^27^

Furthermore, although there are clinical differences between GBD and HT, some overlap may occur between the two entities, especially in individuals with hyperthyroidism, where it may be unclear whether the diagnosis is GBD or Hashitoxicosis. In these cases, the presence of Abs against the thyrotropin receptor (TRAb), mainly in patients with clinical findings, such as exophthalmos, pretibial myxedema, overt hyperthyroidism, diffuse goiter with a “thyroid hell” pattern on Doppler US, and increased uptake on thyroid RI, points more clearly to GBD.^28^,^29^

On the other hand, PTL is a rare subtype of thyroid lymphoma, accounting for approximately one-quarter of all primary lymphomas of the gland. PTL is almost exclusively non-Hodgkin B-cell lymphoma (BCL). The most common subtype is diffuse large BCL, representing 50–70% of PTLs, followed by mucosa-associated lymphoid tissue lymphoma, which accounts for 10–50%.^30^,^31^

Previous reports have shown that patients with HT have a 67-fold higher risk of developing PTL compared to their counterparts with colloid goiter. As many as 90% of PTL cases have a positive history of thyroid autoimmunity and are more commonly associated with mucosa-lymphoid tissue lymphomas than with other subtypes.^31^

PTL typically occurs in individuals over 60 years of age and presents with accelerated glandular growth (generally over a period of months), hard consistency, and compressive symptoms. US findings may reveal a diffuse, homogeneous, pseudocystic hypoechoic pattern, or the presence of a solid, hypervascular, hypoechoic mass, usually without calcifications. These components are rarely observed simultaneously in HT, which usually presents as a diffuse goiter with rapid growth in young individuals, whereas in older adults, the disease tends to progress more gradually.^30^-^32^

Another differential diagnosis of HT is OA. In this context, the diagnosis is usually established during the evaluation of a nodular thyroid condition, as this tumor does not typically cause changes in thyroid function. On US, the presence of a well-circumscribed lesion within a normal sonographic background and with firm consistency raises suspicion for a tumor, whereas a more hypoechoic lesion is less likely to be neoplastic in nature.^33^,^34^

In this context, it is necessary to analyze other important parameters, such as changes in the size of the nodule and the extranodular sonographic appearance of the thyroid. The positivity of thyroid Abs points more toward HT; however, their presence does not exclude the possibility of an OA. In addition, nodular “low uptake” may be observed on RI of the thyroid.^33^-^35^

RT is a type of thyroiditis with an etiology that is not completely understood, although it has been associated with an idiopathic disease (inflammatory fibrosclerosis), characterized by the presence of mediastinal and retroperitoneal fibrosis, sclerosing cholangitis, and inflammatory pseudotumor of the orbit. A fibrotic component may also be found in the gonadal, pituitary, lacrimal, and orbital tissues, among others; these findings have been observed in about one-third of RT cases.^36^

It has also been suggested that RT results from an autoimmune process, based on the presence of thyroid Abs in up to 65% of patients. In addition, the disease has been proposed as part of a systemic IgG4-related disorder, with a clinical presentation typically characterized as inflammatory, compressive, and painless.^36^,^37^

The neck US may show a hypovascular, hypoechoic mass involving extrathyroidal tissues and, occasionally, the carotid vessels. When available, elastography demonstrates stiff inflammatory tissues consistent with fibrosis. On computed tomography, the mass often appears hypodense and does not enhance with contrast. Positron emission tomography exhibits intense uptake in areas of inflammation and is particularly useful for identifying distant fibrotic involvement that may occur in association with RT.^37^,^38^

Diagnostic criteria for RT have been proposed and include: (i) Extrathyroidal extension of the inflammatory process; (ii) presence of occlusive phlebitis; (iii) absence of granulomas, giant cells, lymphoid follicles, or oncocytes; (iv) absence of thyroid malignancies.^36^-^38^

Finally, a differential diagnosis between HT with PTC should be considered. Clinically, PTC typically arises from a nodular thyroid condition, and its presence is suspected based on US findings, together with other clinical parameters, such as accelerated nodule growth, dysphonia, stony consistency, extension to adjacent tissues, and adenomegaly, among others. There are no definitive clinical criteria that distinguish HT from PTC; however, a possible association between autoimmune thyroid disease, hypothyroidism, and PTC has been described.^24^,^39^

The clinical, sonographic, and RI features of HT, DQT, GBD, PTL, RT, and PTC are summarized in Table 1.

**Table 1:** Clinical, etiological, laboratory, and imaging characteristics supporting the differential diagnosis of Hashimoto’s thyroiditis from other thyroid diseases^19^-^39^

Characteristics	HT	DQT	GBD	PTL	OA	RT	PTC
Age at onset (years)	All ages, peak 30–50	20–60	20–50	50–70	40–60	30–50	40–60
Sex ratio (F: M)	4–10:1	4–5:1	8–10:1	8:1	2–3:1	8–9:1	4–9:1
Etiology	Autoimmune	Usually infectious (post-viral)	Autoimmune	Likely autoimmune (related to HT)	Multifactorial	Autoimmune/IgG4- associated	Multifactorial
Thyroid function	Euthyroidism or hypothyroidism (hyperthyroidism may be present in the early stages, that is., Hashitoxicosis)	Biphasic pattern (initial hyperthyroidism) followed by hypothyroidism, with or without complete recovery	Euthyroidism or hyperthyroidism	Euthyroidism	Euthyroidism	Euthyroidism (with subsequent hypothyroidism if associated with HT)	Usually normal
US	Goiter or thyroid atrophy, hypoechoic, pseudonodular or cystic pattern, with or without fibrosis	Hypoechoic, heterogeneous, hypovascular pattern	Diffuse, hypervascular goiter	In diffuse PTL, a hypoechoic, heterogeneous pattern is found; in non-diffuse PTL, goiter can be found with solid, hypoechoic, well-defined masses with calcifications and vascularization	Well-circumscribed nodular lesion in an ultrasonographically normal fundus, with a hardened consistency	Hypoechoic, hypovascular pattern, with fibrosis extending to adjacent tissues	Nodule (s) solid, with microcalcifications, taller than wide, hypoechoic, with poorly defined borders
Radionuclide imaging of the thyroid (Tc-99 or 123/131-I)	Low uptake or normal uptake	Low uptake	Elevated uptake	Normal uptake	Nodule (s) with low uptake	Low uptake or normal uptake	Nodule (s) with low uptake
TPOAb	90–100%	Maybe present	80%	Maybe Present	Variable	Variable	They may be present
TgAb	80–90%	Maybe present	30–60%	Maybe Present	Variable	Variable	They may be present
TRAb	~10%	Negative	90–95%	Negative	Negative	Negative	Negative
CRP and/or ESR	Normal	High	Normal	Normal	Normal	Variable, may be elevated (both)	Normal

Abbreviations: CRP: C-reactive protein; DQT: De Quervain’s thyroiditis; ESR: Erythrocyte sedimentation rate; F: Female; GBD: Graves–Basedow disease; HT: Hashimoto’s thyroiditis; IgG4: Immunoglobulin G4; OA: oncocytic adenoma; M: Male; PTC: papillary thyroid carcinoma; PTL: primary thyroid lymphoma; RT: Riedel’s thyroiditis; TgAb: Antibody against thyroglobulin receptor; TPOAb: Antibody against thyroid peroxidase receptor; TRAb: Antibody against thyrotropin receptor; US: Ultrasound.

## 7. Histopathological findings in DQT

The macroscopic features of DQT may include a firm, white-brown thyroid, with or without nodules or pseudonodules. Microscopically, the histological pattern is typically multiform, with areas of thyroid follicle destruction due to granulomatous infiltration composed of lymphocytes, macrophages, and occasional multinucleated giant cells.^40^,^41^

Destruction of the thyroid follicles leads to colloid extravasation and loss. Areas of fibrosis and acute inflammation (evidenced by the presence of polymorphonuclear neutrophils) may also be observed, along with the occasional formation of microabscesses. These findings generally occur during the acute phases of the disease. In later stages, this infiltrate is replaced by a chronic inflammatory infiltrate.^40^

In the regenerative phase, the thyroid follicles are mostly restored. They vary in size and are embedded within a stroma showing mild-to-moderate fibrosis. In more severe cases, these changes may progress to replacement of the damaged follicles by scar-like structures, devoid of colloid and lacking recognizable follicular cells. Multinucleated giant cells, along with histiocytes and plasma cells, are also present (a finding that supports the designation of this condition as “granulomatous thyroiditis”).^21^,^40^,^41^

**Figure 10:**
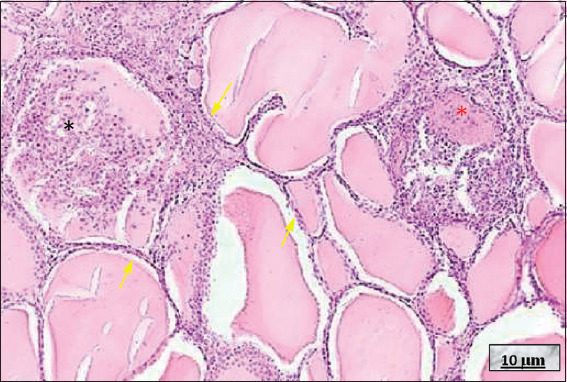
De Quervain’s thyroiditis. Image at 10× magnification, hematoxylin and eosin-stained; scale bar = 10 μm. Note that the dilated thyroid follicles lined by slightly flattened thyrocytes (yellow arrows). In the upper right, a granuloma formed by epithelioid cells and a multinucleated giant cell is observed (red asterisk). In the upper left, abundant foamy histiocytes are visible in the colloid (black asterisk). Source: Authors’ data.

Interfollicular fibrosis and interstitial inflammatory cell reactions may also be observed (Figure 10).

## 8. Histopathological findings in GBD

Macroscopically, in its classic florid form, GBD presents with diffuse gland growth, a prominent vascular pattern on the surface, and a weight ranging from 50 to 150 g. The cut surface appears reddish, resembling skeletal muscle, and the tissue is spongy or firm in consistency.^9^,^20^,^42^,^43^

Microscopically, glandular hyperplasia is the most common histological manifestation of GBD. Diffuse epithelial hyperplasia is typically observed, often forming papillary projections into the lumen of the follicles, which contain little colloid with a scalloped appearance.^9^,^42^,^43^

**Figure 11:**
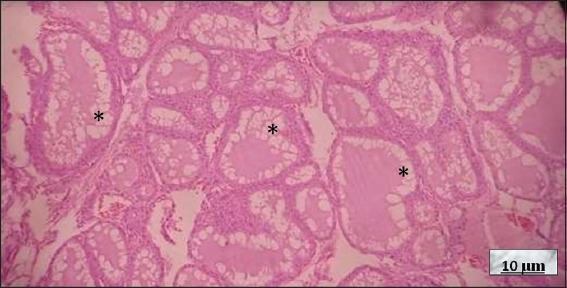
Graves–Basedow disease (GBD). Image at 10× magnification, hematoxylin and eosin-stained; scale bar = 10 μm. Note the predominantly medium-sized thyroid follicles lined with cubic thyrocytes. Within the follicles, pink, scalloped colloid, both pale and intense (black asterisk), is observed, characteristic of long-standing, untreated GBD. Source: Authors’ data.

The papillae have a fibrovascular stem and are lined by columnar or cuboidal epithelium with round or oval basal nuclei. The follicles are generally small and exhibit scalloped colloid, indicating a marked increase in glandular activity^42^,^43^ (depicted in Figure 11).

**Figure 12:**
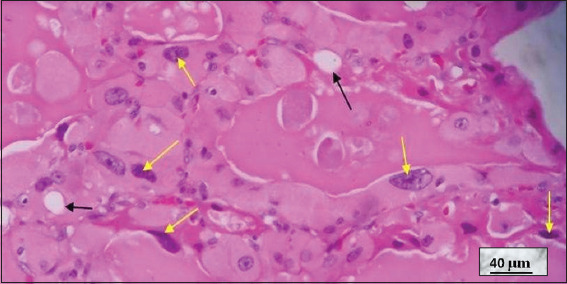
Graves–Basedow disease in a patient treated with thionamides. Image at 40× magnification, hematoxylin and eosin-stained; scale bar = 40 μm. Note the slightly distorted thyroid follicles lined by cuboidal or slightly flattened epithelium, with evident nuclear atypia (yellow arrows) and a pink and pale, slightly scalloped colloid (black arrows). The nuclear changes are attributable to thionamide treatment. Source: Authors’ data.

Glandular fibrosis is usually absent or minimal. However, in patients treated with thionamides, epithelial hyperplasia tends to regress, follicles return to normal size, and colloid volume inside the follicles increases. In contrast, treatment with radioactive iodine can induce fibrotic changes and lymphocytic infiltration, often diffusely distributed. Nuclear atypia – ranging from mild-to-severe – may also occur, with the presence of large, hyperchromatic, irregular nuclei.^9^,^42^,^43^ (Figure 12).

If hyperplasia persists for many months or years, oxyphilic/oncocytic metaplasia may develop, accompanied by irregular stromal proliferation and nodularity, comparable to that observed in euthyroid goiter. If the disease regresses spontaneously or with thionamide therapy, the involution may be either complete or irregular, with residual foci of hyperplasia.^23^,^42^,^43^

**Figure 13:**
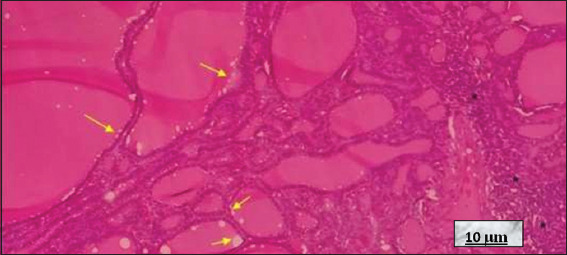
Graves–Basedow disease (GBD) concomitant with Hashimoto’s thyroiditis (HT). Image at 10× magnification, hematoxylin and eosin-stained; scale bar = 10 μm. Toward the right side of the image, a cluster of lymphocytes can be seen extending from the bottom to the top of the image (black asterisks). Toward the central and left parts, there are medium- and large-sized thyroid follicles, lined by cubic thyrocytes (yellow arrows), filled with abundant, intensely eosinophilic colloid. On the colloid, “bubble-shaped” structures or “scalloped colloid” are observed. The scalloped appearance of the colloid, the presence of medium-sized thyroid follicles, cubic follicular cells, and abundant lymphocytes are characteristic findings of short–term GBD coexisting with HT. Source: Authors’ data.

Occasionally, both HT and GBD may coexist in the same patient. These individuals typically have positive thyroid Abs (TPOAb, TgAb, and TRAb) and may alternate between clinical and laboratory manifestations of hypothyroidism and hyperthyroidism. Histopathological features of both diseases may be present concurrently^9^,^23^ (Figure 13).

## 9. Histopathological findings of PTL

As aforementioned, PTL is a rare subgroup of thyroid lymphoma, accounting for one quarter of all primary lymphomas of the gland. PTL shares the same biological origin as HT, which can complicate histological differentiation. Macroscopically, PTL can be unilateral or bilateral, with tissue consistency varying between soft and hard. The surface may appear diffuse, lobulated, and/or multinodular, and areas with both solid and cystic components are frequently observed. Upon sectioning, the surface may be smooth, raised, pale tan, grayish-white, or red, often with a “fish-like” appearance. Extension into the perithyroid adipose tissue or adjacent muscles is common.^31^,^44^,^45^

**Figure 14:**
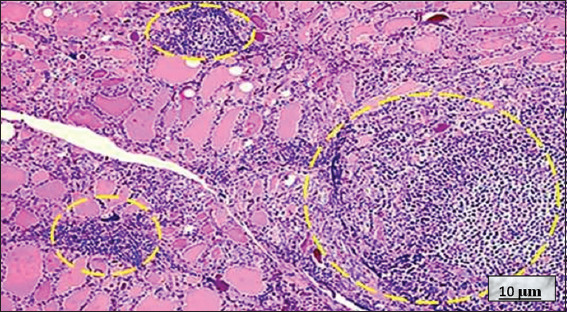
Primary thyroid lymphoma. Image at 10× magnification, with hematoxylin and eosin staining; scale bar = 10 μm. On the right side of the image, a large accumulation of atypical lymphocytes infiltrates the thyroid tissue, “filling the thyroid follicles” and creating the characteristic appearance of “PTL balls” (yellow dotted circle). On the left side of the image, thyroid follicles of different sizes, irregular in shape, and containing colloid are observed. Within these follicles, there is abundant atypical lymphocytic infiltration. Source: Authors’ data.

Microscopically, an extensive lymphocytic infiltrate is observed, with effacement of the normal glandular architecture due to the presence of atypical lymphocytes. The infiltrate consists of lymphoid cells with small, slightly irregular nuclear contours (folded or resembling centrocytes), condensed nuclear chromatin, and barely visible nucleoli. Centroblasts with round and regular nuclear membranes, containing one or two nucleoli, are also present. Lymphoepithelial lesions are typical and have a distinctive appearance of “balls” or rounded masses that fill and distend the lumen of thyroid follicles (often referred to as “PTL balls”).^44^-^46^ Cells with plasmacytoid differentiation are also present (Figure 14).

**Figure 15:**
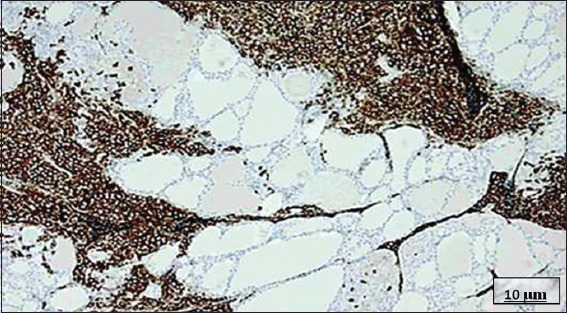
Immunohistochemical image of primary thyroid lymphoma. Image at 10× magnification, with cluster of differentiation (CD) 20 monoclonal antibody staining; scale bar = 10 μm. This reveals strong, diffuse staining, indicating the monoclonality of the lymphocytic infiltrate. In this case, CD20 positivity reveals the presence of a B-cell lymphoma. Source: Authors’ data.

In cases where it is difficult to distinguish between HT and PTL, an immunohistochemical study is essential. The markers that differentiate these entities include lymphoma markers (*e*.*g*., cluster of differentiation [CD] 20, CD79a, BCL2, CD10, BCL6, multiple myeloma oncogene), while in HT, epithelial markers, such as cytokeratin are positive. On the other hand, lymphoid markers, such as CD3 and CD20 will be positive in a mixed manner, ruling out monoclonality of the inflammatory infiltrate that is characteristic of lymphomas^47^,^48^ (Figure 15).

## 10. Histopathological findings of OA

Hürthle cell neoplasms are heterogeneous tumors that arise from follicular cells and are predominantly or exclusively composed of cells exhibiting oncocytic features, also known as oncocytes. Oncocytes microscopically feature an abundant, granular cytoplasm.^49^

Microscopically, the main features of these neoplasms include the presence of oncocytic cells, which possess a large and granular cytoplasm due to abundant mitochondria. These cells may be dispersed or arranged in follicular or papillary patterns, and are often associated with abundant lymphocytes. This latter feature is a confounding variable in the differential diagnosis with HT.^49^,^50^

Hürthle cell neoplasms are defined as tumors composed of more than 75% Hürthle cells. They are classified as OA when histological findings are benign. These include enlarged nuclei with slight atypia, visible nucleoli, wide and granular cytoplasm, and a well-defined, integrated capsule.^33^,^34^,^49^,^50^

**Figure 16:**
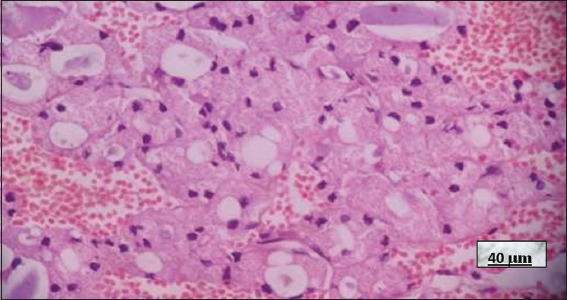
Oncocytic adenoma. Image at 40× magnification, with hematoxylin and eosin staining; scale bar = 40 μm. Large cells are observed with small nuclei, slight anisonucleosis, and mild atypia. The cells have a broad, well-defined, microgranular cytoplasm, and form microfollicles. In this case, the nodule is composed entirely of oncocytic cells. Source: Authors’ data.

**Figure 17:**
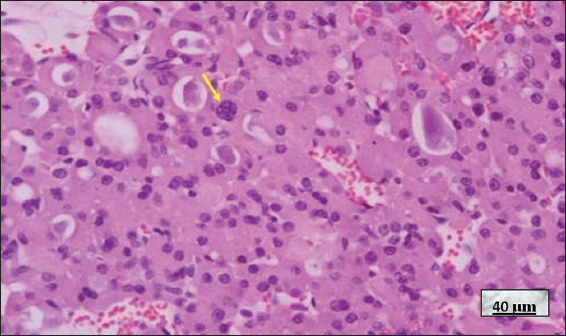
Oncocytic carcinoma. Image in 40× magnification, with hematoxylin and eosin staining; scale bar = 40 μm. Microfollicles are lined by large cells with hyperchromatic nuclei, marked atypia, irregular chromatin, and visible micronucleoli. Some cells are multinucleated (yellow arrow). The cytoplasm is broad and microgranular (denser than in the previous image), and the cells form microfollicles with little or no colloid inside. These cells occupy 100% of the nodule. Source: Authors’ data.

In such benign cases, no recurrence is typically observed following surgical removal. In contrast, oncocytic carcinoma (previously known as Hürthle cell carcinoma) demonstrates histopathological features, such as capsular and/or lymphatic invasion, obvious nuclear atypia, multinucleation, and pleomorphism. This variant exhibits a more aggressive behavior than follicular carcinoma, often showing extrathyroidal extension, local recurrence, or lymph node metastasis. Tumors with a smaller number of Hürthle cells can be classified as variants of papillary or follicular carcinoma^33^,^49^,^50^ (Figures 16 and 17).

## 11. Histopathological findings of RT

Macroscopic features of RT include extensive thyroid fibrosis. The cut surfaces are tan-gray, avascular, stony, and without apparent lobulation. Fibrosis frequently extends into the surrounding soft tissues. Clinically, this appearance may correspond to a hard/stony thyroid gland on palpation and can be accompanied by dysphonia, dysphagia, and stridor, with vocal cord paralysis due to involvement of the recurrent laryngeal nerve. In some cases, large arterial and/or venous vessels of the neck may also be affected.^51^,^52^

**Figure 18:**
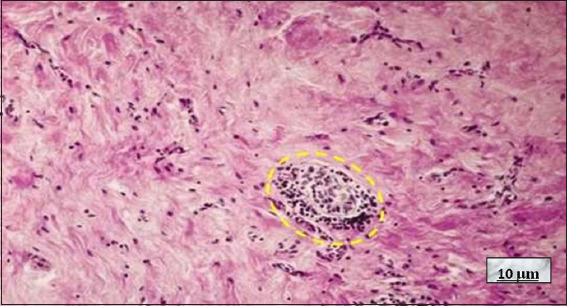
Riedel’s thyroiditis. Image at 10× magnification, hematoxylin and eosin-stained; scale bar = 10 μm. Note the fibrotic background with scattered lymphocytes. In the center, a residual thyroid follicle is observed surrounded by lymphocytes (yellow dotted circle). Source: Authors’ data.

Microscopically, thyroid tissue (or adjacent tissue) may be replaced by extensive fibrosis and an inflammatory infiltrate, predominantly composed of a mixed population of lymphocytes, plasma cells, and eosinophils. Small amounts of colloid may be found in a dense matrix of hyalinized connective tissue, with small entrapped thyroid follicles. A characteristic feature is inflammation involving venous vascular structures.^51^,^52^ Importantly, the absence of granulomatous or malignant tissue is a key criterion (Figure 18).

A particularly challenging differential is with sarcomas of the thyroid region or the paucicellular variant of anaplastic thyroid carcinoma.^53^ Several histopathological characteristics that help distinguish HT from other diseases are summarized in Table 2.

**Table 2:** Differential diagnosis of Hashimoto’s thyroiditis from a histopathological perspective^40^-^53^

Characteristics	HT	DQT	GBD	PTL	Hürthle cell tumor	RT	PTC
Macroscopic global findings	Fibrotic parenchyma with lymphoid appearance	Firm, hard gland; whitish	Spongy and firm consistency; reddish parenchyma	Diffuse, lobulated, and/or multinodular parenchyma; pale cinnamon, grayish-white, or red with a “fish flesh” appearance	Nodular/multinodular gland, with or without hemorrhage or cystic degeneration	Severely fibrotic parenchyma with extension to adjacent tissues	Nodule (s) of variable size, indurated, whitish, or yellowish in color
Global microscopic findings	Lymphoid aggregation, thyroid follicular destruction, and tissue fibrosis; prominent germinal centers with small lymphocytes, centrocytes, centroblasts, plasma cells, and occasional immunoblasts	Multinucleated giant cells, lymphocytes, plasma cells, and histiocytes; Microabscesses and follicular epithelial cell destruction with colloid extravasation and depletion	Glandular hyperplasia with papillary projections into the follicular lumen; papillae with fibrovascular stalks, lined by columnar epithelium with round or oval basal nuclei	Extensive lymphocytic infiltration with architectural effacement by atypical cells; lymphoepithelial lesions forming “PTL balls” that distend the lumen of thyroid follicles	Oncocytic cells with broad and granular cytoplasm, arranged in follicular or papillary patterns, associated with abundant lymphocytes; malignant features include marked cellular atypia, atypical mitoses, lymphovascular invasion, and nodule capsular invasion	Diffuse sclerotic parenchyma, follicular obliteration, extensive inflammatory infiltration; vasculitis and a thrombus-like effect may be present	Nuclear inclusions, orphan Annie nuclei, nuclear grooves, papillary projections, microcalcifications, psammoma bodies; multinucleated giant cells may also be present within the tumor

Abbreviations: DQT: De Quervain’s thyroiditis; GBD: Graves–Basedow disease; HT: Hashimoto’s thyroiditis; PTC: papillary thyroid carcinoma; PTL: primary thyroid lymphoma; RT: Riedel’s thyroiditis.

## 12. Conclusion

HT is an organ-specific autoimmune disease characterized by the presence of thyroid Abs, particularly TgAb and TPOAb. Histopathologically, HT is hallmarked by thyroid infiltration with inflammatory cells, tissue destruction, and frequent fibrosis, which increases the risk of overt hypothyroidism. The main differential diagnoses of HT include, among others, DQT, GBD, PTL, OA, RT, and PTC. Diagnostic confirmation is based on clinical, laboratory, imaging, and, ultimately, histopathological findings.

This review highlights several important clinical implications. For example, it summarizes and classifies the main clinical, etiological, laboratory, imaging, microscopic, and macroscopic features of HT and the key conditions to consider in its differential diagnosis. This structured process may help clinicians gain greater conceptual clarity and reduce uncertainties in the diagnostic approach to this disease.

## Data Availability

All data generated in this study are included in the article. Further inquiries can be directed to the corresponding author.
